# Genetic Variants of Neurotransmitter-Related Genes and miRNAs in Egyptian Autistic Patients

**DOI:** 10.1155/2013/670621

**Published:** 2013-12-23

**Authors:** Ahmed M. Salem, Samira Ismail, Waheba A. Zarouk, Olwya Abdul Baky, Ahmed A. Sayed, Sawsan Abd El-Hamid, Sohair Salem

**Affiliations:** ^1^Department of Biochemistry, Ain Shams University, Cairo, Egypt; ^2^Department of Clinical Genetics, National Research Centre, Giza, Egypt; ^3^Department of Molecular Genetics, National Research Centre, Giza, Egypt; ^4^Department of Child Psychiatry, Ain Shams University, Cairo, Egypt

## Abstract

Autism is a neurodevelopmental disorder with indisputable evidence for a genetic component. This work studied the association of autism with genetic variations in neurotransmitter-related genes, including *MAOA uVNTR, MAOB* rs1799836, and *DRD2 TaqI A* in 53 autistic patients and 30 healthy individuals. The study also analyzed sequence variations of *miR-431* and *miR-21*. *MAOA uVNTR* was genotyped by PCR, *MAOB* and *DRD2* polymorphisms were analyzed by PCR-based RFLP, and *miR-431* and *miR-21* were sequenced. Low expressing allele of *MAOA uVNTR* was frequently higher in female patients compared to that in controls (OR = 2.25). *MAOB* G allele frequency was more significantly increased in autistic patients than in controls (*P* < 0.001 for both males and females). *DRD2 A1+* genotype increased autism risk (OR = 5.1). Severity of autism tends to be slightly affected by *MAOA/B* genotype. Plasma *MAOB* activity was significantly reduced in G than in A allele carrying males. There was no significant difference in patients and maternal plasma *MAOA/B* activity compared to controls. Neither mutations nor SNPs in *miR-431* and *miR-21* were found among studied patients. This study threw light on some neurotransmitter-related genes suggesting their potential role in Autism pathogenesis that warrants further studies and much consideration.

## 1. Introduction

Autism is a neurodevelopmental disorder characterized by disturbances in social interactions, language, and communication, as well as by the presence of stereotyped behaviors and restricted interests. The population prevalence of autism is approximately 15–20 in 10,000 [1]. The assessment of candidate genes for the identification of susceptibility loci in autism is a common molecular strategy. Autism susceptibility loci have been identified on almost every chromosome but no single chromosomal location, however, has been found to be highly significant [2].

The current study investigated genetic variations of two classes of genes, neurotransmission related genes (*MAOA*/*B and DRD2*) and miRNAs (*miR-431* and *miR-21*). *MAOA*/*B* were chosen due to their role in enzymatic degradation. *MAO* has been considered a candidate gene for autism susceptibility based not only on its function, but also on its location on the X chromosome; this is due to the skewed sex ratio in autism (4 males : 1 female) [3]. An association between patients and maternal *MAOA uVNTR* and more severe symptoms of autism spectrum disorders (ASD) was reported [4, 5]. *DRD2* gene is related to receptors in postsynaptic component. Functional polymorphisms which affect receptor availability, either post- or presynaptically, may contribute to the impairments found in individuals with autism [6]. The role of the *DRD2 *gene in autism susceptibility was suggested by the fact that antipsychotic medications, which prevent dopamine D2 receptor activation, improve the core symptoms of ASDs [7].

Monoamine oxidase A (*MAOA*) gene contains a 30 bp variable number of tandem repeats (*VNTR*) in the promoter (1.2 kb upstream) region, termed *uVNTR MAOA*. Although alleles with 2, 3, 3.5, 4, 4.5, 5, and 6 repeats have been reported, variants with 3 and 4 repeats constitute more than 97% of the alleles in all reported control samples. Importantly, 3.5 or 4 copies (high activity alleles) of the repeat sequence are transcribed 2–10 times more efficiently than those with 3 or 5 copies (low activity alleles) of the repeat [8]. Monoamine oxidase B (*MAOB*) contains a single-stranded conformational polymorphism in intron 13, a transitional conversion of adenine (*A*) to guanine (*G*) at a position 36 base pairs (bp) upstream from the 5′end of exon 14 [9]. The human dopamine D2 receptor (*DRD2*) gene contains a *TaqI A* restriction fragment length polymorphism in a noncoding region downstream of the 3′untranslated region at position 32806 of the *DRD2* locus, creating the A1 and A2 alleles [10].

MicroRNAs (miRNAs) are small, 21–25 nucleotide, and nonprotein-coding RNAs that modulate gene expression via the RNA interference pathway [11]. Hypothetically, mutations in the pri- and pre-miRNA regions of miRNA genes could affect processing of the precursor to the mature form of miRNA, resulting in aberrant expression of miRNAs. Altered miRNA expression levels are observed in postmortem cerebellar cortex from autism patients, including* miR-431* and *miR-21* [12]. These findings suggested that sequence variations in *miR-431* and *miR-21* may contribute to autism spectrum phenotype.

This work aims to study the association between autism and *MAOA uVNTR*, *MAOB* rs1799836, and *DRD2 TaqI A*. Also the study analyzed genomic sequence variations of *miR-431* and *miR-21*.

## 2. Subjects

The study included 53 autistic patients (39 males and 14 females) with mean age 5.2 (±2.5) who met the diagnostic criteria of Autism according to DSM-IV: 48 mothers (35 males and 13 females) and 30 healthy age-matched individuals as control group (18 males and 12 females) with mean age 4.5 (±2). All subjects are Egyptians. 48 mothers were analyzed for *MAOA* and 43 for *DRD2*. Patients who were recruited among patients referred to the clinic of National Research Centre. Medical history of patients was investigated including prenatal and postnatal history, onset and progress of symptoms, pedigree construction up to three generations with particular emphasis on consanguinity, and similarly affected family members. Severity of Autism was evaluated according to Childhood Autism Rating Scale (CARS). Patients with associated psychiatric disorder were excluded from the study. The study protocol was approved by the Medical Research Ethics Committee of National Research Centre.

## 3. Method

### 3.1. Genotyping

Genomic DNA was extracted from whole blood samples using standard phenol chloroform protocol [13]. PCR was performed for *MAOA/B*, and *DRD2 *in a reaction mixture contained 300 ng DNA, 3 *μ*L of 5X *Taq* buffer, 3 *μ*L of 0.25 Mm of dNTPs mix, 1.5 *μ*L of 20 pmol of each primer, and 1 U of *Taq* polymerase in a total volume of 25 *μ*L. The cycling reaction was performed under the following conditions: initial denaturation at 95°C for 5 min, followed by 35 cycles each 95°C for 40 Sec, primer annealing according to each primer (Table 1) for 30 Sec, 72°C for 50 Sec, and a final elongation for 7 min at 72°C. PCR products were separated by 2% agarose gel electrophoresis and visualized under UV transilluminator.

30 bp *MAOA uVNTR* polymorphism was analyzed by different fragment sizes [14] which were determined by comparisons to molecular length standards and confirmed by software analysis (lab image). *MAOB* polymorphism was analyzed as previously described [9]; 10 *μ*L of the PCR product was digested with restriction enzyme Tsp45I and products were run on a 3% agarose gel. The *DRD2TaqI A* polymorphism was analyzed as described by Wang et al. [15] by digestion with restriction enzyme *TaqI A*.

Sequence and genomic location data of *miR-431* and *miR-21* were obtained from the Sanger Institute miRBase (http://microrna.sanger.ac.uk/sequences/index.shtml), and the flanking regions of miRNA genes were obtained by BLAT analysis (http://genome.ucsc.edu/cgi-bin/hgBlat?command=start). Primers used were designed using primer3 (http://biotools.umassmed.edu/bioapps/primer3_www.cgi). PCR was performed and then sequencing was carried out to screen SNPs or mutations.

### 3.2. Plasma *MAO* Activity

*MAO A/B* activity in plasma was measured by *MAO-Glo* assay from promega according to the manufacturer.

### 3.3. Statistical Analysis

Since *MAOA* and *MAOB* are X linked, data were analyzed separately for each gender. Maternal genotyping was performed only for *MAOA* and *DRD2*. SNPstats online software was used to analyze genotype and allele frequencies, odd ratio (OR), and Hardy Weinberg equilibrium. *MAOA uVNTR* alleles were classified as low expressing allele (3 and 5 repeats) and high expressing allele (4 repeats). Autism severity was assessed by CARS as mild, moderate, and sever. SPSS statistical software used *t*-test to compare plasma *MAOA* and *MAOB* activity between autistic patients and controls. One way ANOVA test was used to compare the enzyme activity between different genotypes. Males are hemizygous for *MAOA/B* while females may be homo- or heterozygous so genotype frequency was calculated only for females and enzyme activity was analysed for each genotype.

## 4. Results

Male-to-female ratio in our study was ~3 : 1; males (73.6% males and 26.4% females). Parental consanguinity represented 34%, and about 19% of patients had similarly affected family members. No significant association was found between severity of Autism and either gender or consanguinity (*P* = 0.622 and 0.248 resp.). Significant association was detected between severity and the presence of similar affected family members, as 14 mild cases (100%) had negative family history of the disease (*P* = 0.035).

### 4.1. *MAOA uVNTR*

**Figure 1:**
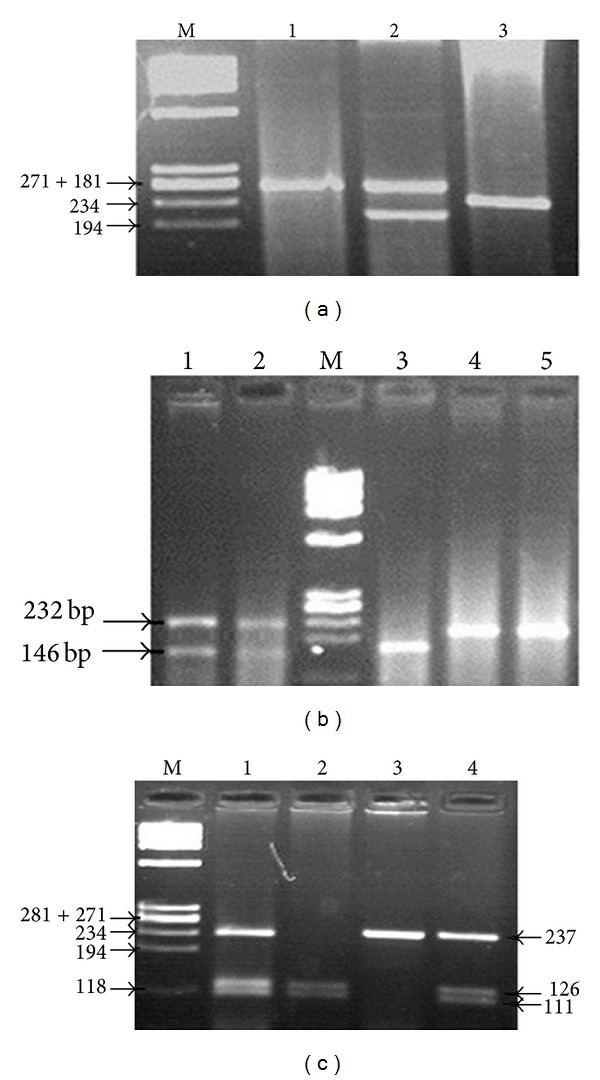
Genotyping of *MAOA*/*B* and *DRD2*. (a) Different *VNTRs* of *MAOA*. M indicates DNA marker (*φ*x174). Lane 1 indicate 5 tandem repeats at 269 bp. Lane 2 indicates 3 and 5 tandem repeats at 209 and 269 bp. Lane 3 indicates 4 tandem repeats at 239. (b) PCR-based RFLP of *MAOB*. M is DNA marker (*φ*x174). Lanes 1 and 2 indicate AG genotype at 232 and 146 bp (86 bp not shown). Lane 3 indicates AA genotype at 146 bp. Lanes 4 and 5 indicate GG genotype at 232 bp. (c) PCR-based RFLP of *DRD2*. Lanes 1 and 4 indicate A1A2 genotype at 237, 126, and 111 bp. Lane 2 indicates A2A2 genotype at 126 and 111. Lane 3 indicates A1A1 genotype at 237 bp.

The 30 bp-repeat polymorphism showed three alleles: 3 repeats: 209 bp; 4-repeat: 239 bp; and 5-repeat: 269 bp (Figure 1). *MAOA uVNTR *was classified in males as low expressing allele (3 repeats) or high expressing allele (4 repeats); the low and high alleles are equally distributed between male cases and controls (OR = 1). Female genotypes were classified into low/low (3/3, 3/5), high/high (4/4), and low/high (3/4) distributed as follows: 21.4%, 35.7%, and 42.9%, respectively. The low/high genotype was frequently higher in female cases than in controls (OR = 3.75, and *P* = 0.14). Low expressing allele was presented in high frequency in female cases than in controls (OR = 2.25, *P* = 0.17) (Table 2).

Maternal low expressing allele was presented in high frequency than controls (OR = 1.29 and 1.59 for male and female mothers resp.) (Table 2). The genotype low/high was more common in mothers than controls (OR = 2.95 and 5.8 for male and female mothers resp.). The genotype frequencies of *MAOA uVNTR* for females, mothers, and controls were all in Hardy-Weinberg equilibrium (*P* = 0.62, 1, and 0.09 resp.). Patients carrying low expressing allele tend to be severely autistic rather than mild to moderate (OR = 2.24 and 1.57 for male and female patients, resp.) (Table 3).

### 4.2. *MAOB* Polymorphism

Allele A showed two bands of 146 and 86 bp (86 bp band not shown), while allele G was detected as uncleaved 232 bp band (Figure 1). G allele was significantly higher in cases than controls (OR = 34 and 12.5 for male and female cases, resp.; *P* < 0.001) (Table 2). A/A genotype was absent in female cases. G/G genotype was absent in controls and represents 42.9% of females cases. A/G genotype was frequently higher in female cases than in controls (OR = 2.67, *P* = 0.22). Genotypes distribution in female cases and controls were in accordance with Hardy Weinberg equilibrium (*P* = 0.51 and 1 resp.). Cases with G allele slightly tend to be severely autistic rather than mild to moderate (OR = 1.173, 2.00 for males and females resp.) (Table 3).

### 4.3. *DRD2TaqI A* Polymorphism

A1 allele was detected by the presence of uncleaved 237 bp band while A2 allele was detected by cleavage of 237 bp band into 111 and 126 pb fragments (Figure 1). A1 allele was presented in higher frequency in autistic patients than in controls (OR = 1.63, *P* = 0.13). Unlike cases, A2 was more common in mothers than controls (OR = 2.28, *P* = 0.018) (Table 2). By classifying the cases into A1+ (A1A1, A1A2) and A1− (A2A2), the A1+ genotype was significantly higher in cases than in controls (OR = 5.1, *P* = 0.04). A1A1 genotype was more common in cases than controls (OR = 2.11, *P* = 0.21) while A2A2 was significantly increased in mothers (OR = 4.348, *P* = 0.007). Mothers and controls genotypes were in accordance with Hardy Weinberg equilibrium (*P* = 0.46, and 0.065 resp.); in contrast cases genotype was deviated from Hardy Weinberg equilibrium (*P* = 0.0004).

### 4.4. Plasma *MAO* Activity in Autistic Patients

No difference of *MAOA/B* plasma activity in autistic patients and their mothers compared to controls (*P* = 0.927, 0.958, 0.4, and 0.7 for males *MAOA*, *MAOB*, maternal *MAOA*, and maternal *MAOB* respectively; *P* = 0.439, 0.449, 0.907, and 0.6 for females *MAOA*, *MAOB*, maternal *MAOA*, and maternal *MAOB* resp.). *MAOA* activity did not differ between males low and high expressing alleles (*P* = 0.96) nor between females genotypes (*P* = 0.408). *MAOB* activity was significantly reduced in G than in A allele carrying males (*P* = 0.027), while females showed no difference of *MAOB* activity between A/G and G/G genotypes (*P* = 0.82).

### 4.5. *miR-431* and *miR-21* Variations

**Figure 2:**
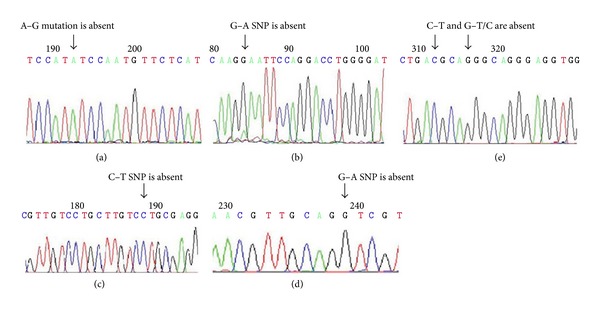
Sequencing chromatogram of *miR-21* and *miR-431*. (a) it shows the absence of A–G mutation downstream the *pre-miR-21*. ((b), (c), (d), and (e)) they show the absence of rs12883709 G–A upstream the *pre-miR-431*, rs76090066 C–T and rs12884005 G–A in the sequence of *pre-miR-431*, rs61993318 C–T, and rs35695758 G–T/C downstream the *pre-miR-431*.

rs12883709 G/A upstream the *pre-miR-431*, rs12884005 G/A and rs76090066 C/T in the sequence of the *pre-miR-431*, rs 61993318 C/T, and rs35695758 G/T/C downstream the *pre-miR-431* were all absent. Also A–G mutation at 29-nt downstream of *pre-miR-21 *was absent. No variations were observed in the amplified sequences (Figure 2).

## 5. Discussion

Twin and family studies provide indisputable evidence for a genetic component in autism. The average concordance for identical twins versus fraternal twins was frequently studied and reported to be 36% versus 3% [16], 64% versus 9% [17], and 60% versus 0% [18]. However, a concordance rate for monozygotic twins of less than 100% indicates that nongenetic factors also play a causal role. The current study showed that about 19% of patients had similar affected family members. Elevated rates of clinical psychiatric disorders, distinct from autism, have been reported among the relatives of individuals with autism, including schizophrenia, anxiety, depression, and social phobias [19]. Parental consanguinity represented 34% of cases in our study; it is higher than that of Saltık and Basgül (21.4%) [20]. However, Datta et al. [21] found that possible parental consanguinity increases the likelihood of autism and behavioral disturbances; many reports did not find a link between consanguinity and autism in Egyptian [22], Saudi [23], and Iranian population [24].

Deletion of *MAOA*/*B* was associated with severe mental retardation and unusual stereotypical behaviors of hand wringing and lip smacking in males [25]. Bortolato et al. [26] found that both *MAOA*/*B* knockout mice displayed neuropathological alterations reminiscent of typical ASD features. Previous study found consistent association between the “low activity” allele of *MAOA* and larger brain volumes for regions of the cortex in children with autism but not in controls. In contrast, the data did not find association of the *MAOA* promoter polymorphism with autism itself [3]. Tassone et al. [27] suggested that functional *MAOA* promoter alleles play a potential role in the male child, the mother, or both in ASD. In this study, no difference in allele frequency of *MAOA* between male autistic patients and controls. However low expressing allele and low/high genotype were more common in female cases and mothers. Severity of autism slightly tends to increase in low expressing allele for both cases and mothers. Previous results found that autistic boys with the low expressing 3-repeat *MAOA* allele had more severe sensory behaviors, arousal regulation problems, aggression, and worse social communication skills than males with the high activity allele and that problems with aggression, as well as with fears and rituals, were modified by the mothers' genotype [5].

The A/G (A644G) noncoding SNP (rs1799836) of *MAOB* is responsible for altered enzyme activity with tissue specificity [28, 29]. This *MAOB* SNP was found to be associated with emotional regulation [30, 31] and Parkinson's disease [32]. Also, it was implicated as risk factor for schizophrenia in a Spanish population [33] and in Han Chinese [34]. So far, no available information was found regarding relation of *MAOB* polymorphism and autism; however, this study highlighted the importance of G allele in both male and female autistic patients. G allele was significantly higher in autistic cases than in controls. Interestingly, G/G genotype was absent in controls, while A/A genotype was absent in female cases. Such findings may suggest its potential role in the impairments found in individuals with autism.

No correspondence was found between the low and high *MAOA* genotype and brain *MAOA* activity in healthy males [35] and in postmortem samples [36]. This study showed that neither *MAOA* nor *MAOB* activity had significant difference in plasma of autistic patients and mothers compared to controls. Partially, results of this study contradict a study on Omani autistic children which showed a significant reduction in the level of plasma *MAOA* activity with nonsignificant change in the level of *MAOB* [37]. No association was found between *MAOA* genotype and plasma enzyme activity. The previous results suggesting that genotype does not have a direct effect on brain enzyme activity [35]. *MAOB* activity was significantly reduced in G allele than in A allele carrying males (*P* = 0.027). The G allele had been associated with lower *MAOB* activity in human brain [38]. In contrast, Garpenstrand et al. [39] found that individuals with the “A-allele” displayed significantly lower platelets enzyme activity than individuals with the “G-allele.” However, Pivac et al. [29] found that platelet *MAOB* activity did not differ between men subjects subdivided into those with A allele or G allele.

Postsynaptic D2 receptors and presynaptic D2 autoreceptors are involved in the dopaminergic (DAergic) modulation of cognitive and emotional processes that are impaired in individuals with autism [40]. Previous results showed that the subjects with one or two A1 alleles had lower *DRD2* density than those without this allele. Therefore, the *DRD2 TaqI A* polymorphism may be one of the important markers for the *DRD2* density and function [41]. Minor allele frequency for the *Taq1 A* ranges from 20% in Caucasians to 44% in other ethnic groups [42]. In this study, A1 allele was predominating in autistic patients than in controls. Unlike cases, A2 was more common in mothers than controls.

A mutation or a single nucleotide polymorphism (SNP) at the miRNA gene region might affect the transcription of pri-miRNA transcripts, the processing of miRNA precursors to mature miRNAs, or miRNA-target interactions [43]. It was suggested that dysregulation of miRNA expression contributes to the observed alterations in gene expression and, in turn, may lead to the pathophysiological conditions underlying autism [2]. A study by Abu-Elneel et al. [12] found that altered miRNA expression levels are observed in postmortem cerebellar cortex from autism patients. Among these dysregulated miRNAs were *miR-431* (14q32.2) and *miR-21 *(17q23.1). rs12883709 G/A, rs12884005 G/A, rs76090066 C/T, rs61993318 C/T, and rs35695758 G/T/C were absent in the current study. Zhu et al. [43] reported A–G mutation at 29-nt downstream of *pre-miR-21* led to a conformational change of the secondary structure close to the stem reaching into the *pre-miR-21* and a relative reduction of the mature *miR-21* expression in vivo. Even though we did not report any of SNPs or mutation in regions amplified, it could be attributed to small number of cases, so a larger number of patients, miRNAs and amplified sequences are recommended.

This study threw light on some neurotransmitter-related genes suggesting their potential role in Autism pathogenesis that warrants further studies and much consideration.

## Figures and Tables

**Table 1:** Primer sequences of PCR.

Gene	Sequence	Product size (pb)	Annealing temperature (°C)
*MAOA *			
Forward	5′-CCCAGGCTGCTCCAGAAAC-3′	209, 239, or 269	52
Reverse	5′-GGACCTGGGCAGTTGTGC-3′		
*MAOB *			
Forward	5′-GGAACCTCTTATACCACAGG-3′	232	58
Reverse	5′-GACTGCCAGATTTCATCCTC-3′		
*DRD2 *			
Forward	5′-CCTTCCTGAGTGTCATCAAC-3′	237	54
Reverse	5′-ACGGCTCCTTGCCCTCTAG-3′		
*miR-431 *			
Forward	5′-GCCTGTAGATCAGGGTCAGG-3′	401	58
Reverse	5′-GACGCTGTGTGAGTTCTTCG-3′		
*miR-21 *			
Forward	5′-GCCAGAAATGCCTGGGTTT-3′	305	54
Reverse	5′-CAAAAGACTCTAAGTGCCACCA-3′		

**Table 2:** Allele frequencies *MAOA uVNTR; MAOB* rs1799836 and *DRD2 Taq1A*.

	Males				Females			
	Cases *n* (%)	Controls *n* (%)	OR (95% CI)	*P* value	Cases *n* (%)	Controls *n* (%)	OR (95% CI)	*P* value
*MAOA uVNTR *								
Low expressing allele	13 (33.3)	6 (33.3)	1 (0.31–3.27)	—	12 (43)	6 (25)	*2.25 (0.69–7.39) *	0.17
High expressing allele	26 (66.7)	12 (66 .7)			16 (57)	18 (75)	0.044 (0.14–1.46)	
Maternal *MAOA uVNTR *								
Low expressing allele	21 (30)	6 (25)	*1.29 (0.45–3.7) *	0.64	9 (35)	6 (25)	*1.59 (0.47–5.42) *	0.46
High expressing allele	49 (70)	18 (75)	0.78 (0.27–2.24)		17 (65)	18 (75)	0.63 (0.18–2.15)	
*MAOB* rs1799836								
G allele	26 (66.7)	1 (5.6)	*34 (4.06–284.347) *	*<0.001 *	20 (71)	4 (17)	*12.5 (3.24–48.26) *	*<0.001 *
A allele	13 (33.3)	17 (94.4)	0.03 (0.00–0.25)		8 (29)	20 (83)	0.08 (0.02–0.31)	

	Patients				Mothers			
	Cases *n* (%)	Controls *n* (%)	OR (95% CI)	*P* value	Cases *n* (%)	Controls *n* (%)	OR (95% CI)	*P* value

*DRD2 Taq1A *								
A1 allele	64 (60)	29 (48)	*1.63 (0.86–3.09) *	0.13	25 (29)	29 (48)	0.44 (0.22–0.87)	*0.018 *
A2 allele	42 (40)	31 (52)	0.61 (0.32–1.16)		61 (71)	31 (52)	*2.28 (1.15–4.54) *	

*P* < 0.05 is significant; OR: odd ratio; 95%; CI: 95% confidence interval.

**Table 3:** Relation between *MAOA/B* polymorphisms and severity of autistic patients.

	Males				Females			
	Severe *n* (%)	Mild and moderate *n* (%)	OR	*P* value	Severe *n* (%)	Mild and moderate *n* (%)	OR	*P* value
*MAOA uVNTR *								
Low expressing allele	7 (43.8)	6 (26.1)	*2.24 *	0.25	5 (50)	7 (39)	*1.57 *	0.57
High expressing allele	9 (56.3)	17 (73.9)	0.45		5 (50)	11 (61)	0.64	
*MAOB* rs1799836								
G allele	11 (68.8)	15 (56.2)	*1.173 *	0.82	8 (80)	12 (66.7)	*2.00 *	0.45
A allele	5 (31.2)	8 (34.8	0.85		2 (20)	6 (33.3)	0.50	
